# Out-of-hours discharge from the ICU: defining the out-of-hours period and its effect on mortality

**DOI:** 10.1186/cc11119

**Published:** 2012-03-20

**Authors:** YL Bramma, R Allan, R Sundaram

**Affiliations:** 1 Royal Alexandra Hospital, Paisley, UK

## Introduction

Out-of-hours discharge from the ICU is associated with increased mortality. In Scotland, approximately 15% of discharges occur out of hours [1]. The aim of this study was to determine the reasons behind out-of-hours discharges in our hospital and the effect this has on mortality.

## Methods

We carried out a retrospective analysis of all patients admitted to our ICU over a 3-year period. Patients who died during their ICU stay, patients <16 years, patients transferred to another ICU, and those with missing data were excluded. Data collected: patient demographics, APACHE II score, time of discharge from the ICU, reason for out-of-hours discharge, and hospital mortality. The out-of-hours period was defined as per the Scottish Intensive Care Society (SICS) as 20:00 to 07:59 hours, then later re-defined as 17:00 to 07:59 hours.

## Results

A total of 766 patients were included: 607 discharged between 08:00 and 19:59 hours, 159 discharged between 20:00 and 07:59 hours. Data are expressed as mean values (SD) or percentages, 'in hours' versus 'out of hours'. Both groups were similar: age 51.9 (18.1) versus 54.0 (17.7) years, males 48.9% versus 50.9%, APACHE II score 15.8 (8.7) versus 17.4 (8.0). Hospital mortality following ICU discharge was 9.9% (55/607 deaths) versus 10.0% (16/159 deaths), RR 1.11 (95% CI 0.66 to 1.88). Discharge was delayed due to a shortage of ward beds in 28.5% versus 43.4% of cases. No early discharges were recorded. With the out-of-hours period re-defined: 393 patients were discharged between 08:00 and 16:59 hours, 373 between 17:00 and 07:59 hours. Both groups were similar: age 51.0 (18.4) versus 53.8 (17.5) years, males 49.9% versus 48.8%, APACHE II 14.9 (8.7) versus 17.4 (8.2). Hospital mortality was 7.7% (28/393 deaths) versus 11.5% (43/373 deaths), RR 1.62 (95% CI 1.03 to 2.55). Discharge was delayed due to a shortage of ward beds in 22.7% versus 41.0% of cases. ICU step-down is most safely performed when medical staffing levels on the wards are highest. The SICS define the out-of-hours period based on the time of handover to nightshift. For discharges at this time, there was no increase in mortality. In our hospital, evening ward cover is the same as overnight. For an out-of-hours period of 17:00 to 07:59, there was a significant increase in mortality following out-of-hours discharge.

## Conclusion

Our data show increased mortality following ICU step-down in the evening as well as at night. Discharge was most often delayed due to a lack of ward beds. To reduce mortality, efforts must therefore be made to improve bed management and ensure discharge from the ICU before 17:00.
